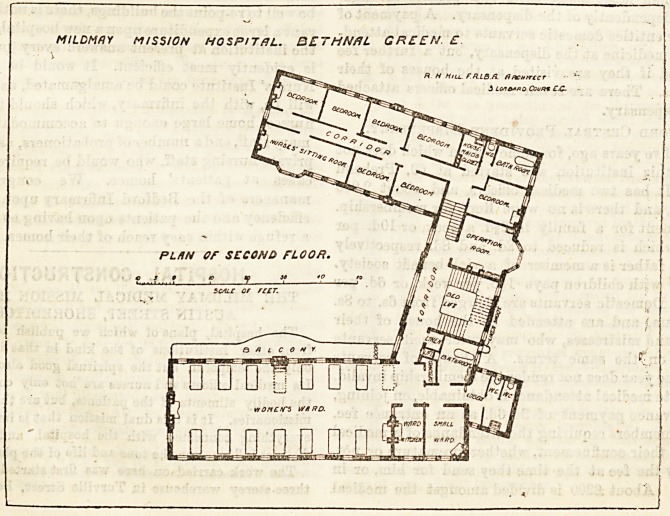# The Mildmay Medical Mission Hospital, Austin Street, Shoreditch

**Published:** 1893-02-04

**Authors:** 


### HOSPITAL CONSTRUCTION.

### THE MILDMAY MEDICAL MISSION HOSPITAL, AUSTIN STREET, SHOREDITCH.

The hospital, plans of which we publish to-day, differs from other institutions of the kind in that its aim is not only the temporal, but the spiritual good also of the sick. Its medical officers and nurses are not only concerned with the bodily ailments of the patients, but are themselves also missionaries. It is this dual mission that is impressed upon everything connected with the hospital, and has a very distinct influence on the tone and life of the place.

The work carried on here was first started in 1876, in a three-storey warehouse in Turville Street, Bethnal Green,

MILDMAY MISSION HOSPITAL.

BETHNAL GREEN. E.

WOMEN'S WARD

WARD KITCHEN

SMALL WARD

with accommodation for some 36 patients. Turville Street forms part of a large insanitary area, which since 1890 has been condemned to well-deserved demolition by the London County Council. Boundary Street area, as it is called, was in fact a network of the worst kind of slums, dark and tortuous courts and alleys teeming with a population of which misery, crime, and disease were the normal conditions. Within a short distance of the old premises the new hospital has been erected, a bright spot amidst its squalid and poverty-stricken surroundings. The new buildings, which were opened on November 11th, 1892, were erected from plans by Mr. R. Hill, F.R.I.B.A., at a cost of some £17,000, including the site.

Entering through the gateway in Austin Street, a short drive leads into an open quadrangle, round three sides of which the buildings are grouped. The central part immediately opposite the entrance is mainly administrative, as also is the wing on the left, while the right wing contains the wards and the out-patient department. The exterior of the building is simple but effective, with its red bricks and terra-cotta, the warm colour contrasting most pleasantly with the dingy, smoke-stained surroundings.

The ground floor of the right wing is occupied by the out-patient department. The large waiting-hall—a bright, cheery-looking room, L-shaped on plan, with a dado of glazed tiles —is also used as a mission hall. From here the patients pass to the consulting rooms, and thence either to the surgery or straight to the waiting room for medicine, which adjoins the dispensary. About 150 patients are daily treated in this department. In all these rooms are pictures, which are fixed on to the surface of the walls with simple painted or distempered borders to serve for frames; a plan which has much to commend it, and one which might well be adopted with advantage in wards. The fittings in the surgery and dispensary are simple, and clean in appearance.

In the left wing are the kitchen offices, with the servants' hall and the staff dining-room. The arrangement for dust and refuse is a good one; leading out of the scullery is a recess open towards the street, in which the rounders for refuse are kept, and from whence the contents are removed by the parish dustmen. Passing up the main staircase, which occupies the centre of the central block, we reach the men's ward on the first floor. The large ward is 60 feet long, 25 feet wide, and 14 feet high, and contains 14 beds in the summer and 13 in the winter, when one bed is removed to make room for a Nautilus stove. The ward is lighted by windows at each side which reach well up to the ceiling. The cubic space per patient is 1,400 feet, and the floor space 107·4 feet. The proportion of wall space to window space appears somewhat small, and the beds are rather too close to the windows for comfort in cold weather. But this is a fault of less importance than if the beds had been coupled; and with a judicious use of screens any danger from draughts can easily be guarded against. The lockers are particularly well devised. The lower part forms a receptacle for clothes, and the lid when shut down becomes a seat; the upper part turns on a pivot and has a hinged flap which forms a bed table, and when let down becomes a back to the seat. It has also shelves for cups and other belongings; at the back is a towel rail. The whole locker is mounted on castors. The ward kitchen is placed between the large ward and a small ward for one bed. Outside the ward is a bath-room and lavatory, and in a projecting wing are two w.c.'s and a sink-room. The slop sinks are of glazed fireclay, but are not by any means of the best form. In addition to the sink there is here a cupboard formed in the thickness of the wall for keeping excreta, and a large glazed sink placed in a similar recess and used for steeping soiled sheets before they are sent downstairs. In the lobby is a cupboard for brooms.

At the further end of the ward a door leads out on to the flat roof over the mission hall, which forms a convenient place where the men can get out into the air and smoke.

The second floor of this wing is precisely similar to the floor below, and contains the women's wards. A balcony runs along the whole length of the ward on the quadrangle side.

On the third floor is the children's ward, with twenty cots. The floor and cubic space in this ward being the same as in the wards below, it follows that the space per patient is less by something over twenty-five per cent. It is very questionable whether children really require a less amount of air than adults; but so great a difference as there is here is surely not warranted. The theory that a child requires less cubic space than an adult is based, presumably, on the fact that a child's lungs are less in capacity than those of an adult; but on the other hand the young lungs work with much greater rapidity than the mature ones, and therefore the difference is not so great as it would seem.

In the corner of the children's ward by the fireplace a deep glazed porcelain sink forms an excellent bath for small children.

The operation room is on the second floor at the left of the staircase. The walls are lined with tiles up to about five feet above the floor. Beyond this there has been no attempt to obtain that condition of absolute asepticism so desirable in an operation room.

In the left wing are the matron's rooms, the resident surgeon's rooms, and the nurses' rooms.

In conclusion, we must add a few words of hearty appreciation of the careful thought for every detail, and the evidences on all hands of skilful and excellent management that are so conspicuous throughout the building. It has rarely been our lot to spend a pleasanter or more instructive afternoon than when, under the guidance of the excellent matron, Miss Goodwyn, we visited every part of this most deserving hospital.

## Figures and Tables

**Figure f1:**
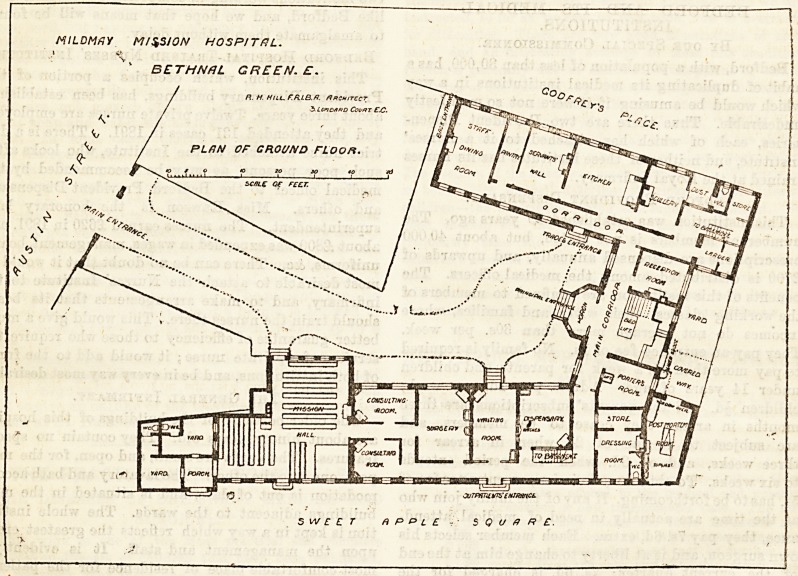


**Figure f2:**